# Occurrence of novel GII.17 and GII.21 norovirus variants in the coastal environment of South Korea in 2015

**DOI:** 10.1371/journal.pone.0172237

**Published:** 2017-02-15

**Authors:** Eung Seo Koo, Man Su Kim, Yong Seon Choi, Kwon-Sam Park, Yong Seok Jeong

**Affiliations:** 1 Department of Biology, College of Sciences, Kyung Hee University, Seoul, Republic of Korea; 2 Department of Food Science and Biotechnology, Kunsan National University, Gunsan, Republic of Korea; University of Malaya, MALAYSIA

## Abstract

Human norovirus (HNoV), a positive-sense RNA virus, is the main causative agent of acute viral gastroenteritis. Multiple pandemic variants of the genogroup II genotype 4 (GII.4) of NoV have attracted great attention from researchers worldwide. However, novel variants of GII.17 have been overtaking those pandemic variants in some areas of East Asia. To investigate the environmental occurrence of GII in South Korea, we collected water samples from coastal streams and a neighboring waste water treatment plant in North Jeolla province (in March, July, and December of 2015). Based on capsid gene region C analysis, four different genotypes (GII.4, GII.13, GII.17, and GII.21) were detected, with much higher prevalence of GII.17 than of GII.4. Additional sequence analyses of the ORF1-ORF2 junction and ORF2 from the water samples revealed that the GII.17 sequences in this study were closely related to the novel strains of GII.P17-GII.17, the main causative variants of the 2014–2015 HNoV outbreak in China and Japan. In addition, the GII.P21-GII.21 variants were identified in this study and they had new amino acid sequence variations in the blockade epitopes of the P2 domain. From these results, we present two important findings: 1) the novel GII.P17-GII.17 variants appeared to be predominant in the study area, and 2) new GII.21 variants have emerged in South Korea.

## Introduction

Human norovirus (HNoV) is a problematic gastroenteritis pathogen found worldwide. This highly infectious virus can be transmitted by various modes, such as direct/indirect contact, waterborne transmission, foodborne transmission, and even airborne transmission in certain settings [1, 2]. HNoV infection and the resulting disease can occur in all age groups; severe gastroenteritis cases are often observed in immunocompromised patients [3–5]. Once infected, the incubation period is typically 24–48 h, and viral shedding in the stool can occur from as short as several days after infection to over two years, depending on the immune status of the patient [6–9].

Norovirus (NoV), a member of the family Caliciviridae is a non-enveloped, single-stranded, positive sense RNA virus with a genome length of approximately 7.5–7.7 kb [10]. The NoV genome contains three open reading frames (ORF1, ORF2, and ORF3; ORF4 is present in murine NoV only) [11]. ORF1 encodes non-structural proteins for genome replication (ns1–7), and ORF2–4 are translated into structural proteins (VP1–2 and VF1) [12]. Because a practical culture system for NoV was only reported recently [13], classification of the NoVs has only been carried out at the genotype level [10]. NoVs have been categorized into six genogroups (GI–GVI), and a recent study also suggested the presence of a seventh genogroup GVII; some of these (i.e., GI, GII, and GIV) are known to infect humans and GII is the main causative agent of viral acute gastroenteritis [10, 14]. Genotypes of NoV have been determined using the polymerase gene alone, the capsid gene alone, or both genes because of the possibility of an ORF1-ORF2 recombination event [15]. Thus far, nine capsid genotypes in GI, 22 genotypes in GII, three genotypes in GIII, and two genotypes each for GIV–VI have been recognized [10]. Of the 22 capsid genotypes in GII, only GII.4 has shown to be associated with global pandemics of gastroenteritis. GII.4 pandemic variants, such as US96, Farmington Hills 2002, Hunter 2004, Den Haag 2006b, New Orleans 2009, and Sydney 2012, have rapidly replaced the former dominant variant, consistent with clinical reports over the last two decades [14]. Recent reports, however, have revealed that novel variants of GII.17 have been overtaking the established GII.4 pandemic variants in some areas of China and Japan [16–19].

South Korea is geographically close to China and Japan. Thus, it is reasonable to assume that the GII.17 variants of China and Japan may have migrated in and become endemic variants. The presence of NoV in human communities can affect the genotype occurrence in adjacent environments including shellfish farms in estuaries [20–22]. In this study, we therefore aimed to determine whether the novel GII.17 variants are prevalent in the coastal environment of South Korea. Focusing on the presence of NoV GII in coastal stream water, treated sewage effluent, and wild-growing clams in a peri-urban estuarine bay of North Jeolla province, we identified two novel variants of HNoVs belonging to either GII.17 or GII.21.

## Materials and methods

### Ethics statement

The sample collection in this study was approved by the Korean Food and Drug Administration (KFDA, Project No. 14162–973). This study did not require additional permission for activities because sample collection was not performed on private land or in protected areas. We confirmed that this study did not involve endangered or protected species.

### Study area, sample collection and preparation

**Fig 1:**
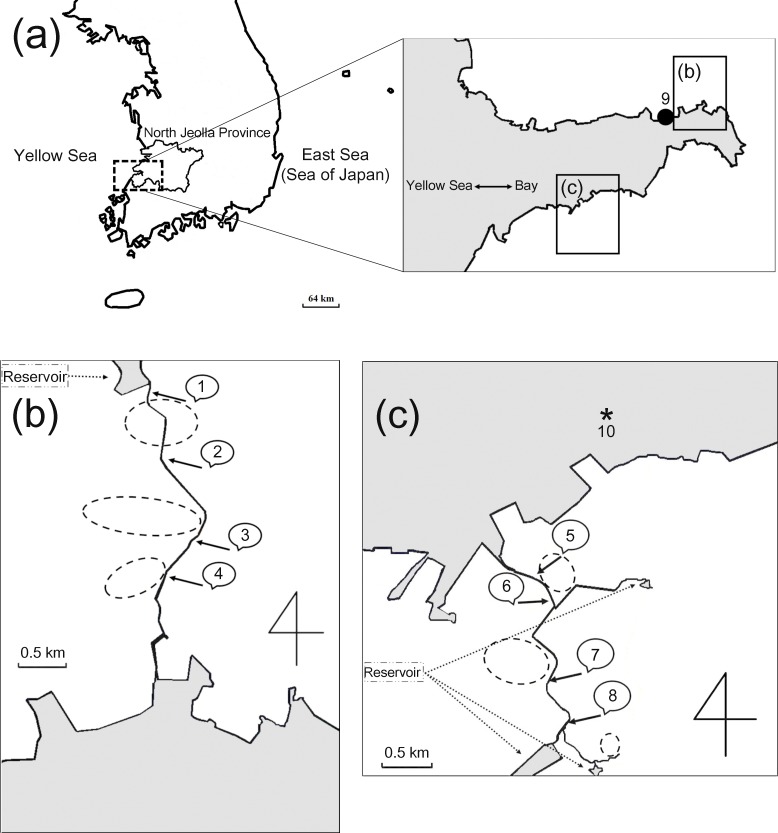
Map of the study area. The top maps in panel (a) show the study area in North Jeolla province, South Korea. Water collection sites (numeric characters) for coastal streams are described in panels (b) and (c). The waste water treatment plant (●, site 9) is shown in the top map. Each water sampling site in the streams was located downstream of drainage for domestic sewage that originated from neighboring dwellings. Circles with dashed lines (- - -) represent temporary locations of dwellings in the vicinity of the streams. The asterisk (*, site 10) in panel (c) indicates the sampling site for wild-growing clams.

Environmental water sample collection was carried out three times (March 21, July 13, and December 28, 2015) at nine sampling points (one for the waste water treatment plant and eight for coastal streams) in an estuarine bay of North Jeolla province, South Korea (Fig 1). In total, 27 environmental water samples, including three treated sewage effluent samples, were collected by passing approximately 100 L of water through NanoCeram filters (Argonide, Sanford, FL, USA) and concentrated according to USEPA method 1615 [23]. The final concentrates were stored at -80°C prior to RNA extraction and analysis. Wild short neck clams (*Tapes philippinarum*) were collected three times (March 21, July 15, and December 28, 2015) in the vicinity of the water sampling site 5 (Fig 1) and stored for less than 24h at 4°C prior to virus isolation.

The isolation process for NoV from short neck clam samples followed the standard method of the KFDA with minor modifications (not published). Briefly, 10 g of digestive gland tissue was obtained from 0.5 kg of the clams and used for subsequent sample processing. The digestive gland tissue was homogenized with 75 mL of ‘buffer A’ (250 mM glycine and 140 mM sodium chloride, pH 7.5), and centrifuged at 10,000 × *g* for 30 min at 4°C to harvest the supernatant. The pellet was resuspended with 75 mL of ‘buffer B’ (250 mM threonine and 140 mM sodium chloride, pH 7.5) and centrifuged at 20,000 × *g* for 30 min at 4°C. The harvested supernatants from the buffer A and buffer B mixtures were combined and precipitated at 4°C for 16 h with 90 mL of ‘buffer C’ (30% polyethylene glycol [PEG] 6000 and 800 mM sodium chloride). The resulting mixture was centrifuged at 10,000 × *g* for 20 min at 4°C, and the PEG 6000 pellet was resuspended with 10 mL of 0.2% Tween 80 in 50 mM Tris buffer. All samples were extracted using an equal volume of chloroform and then centrifuged at 10,000 × *g* for 30 min at 4°C. The supernatant was precipitated again at 4°C for 3 h with an equal volume of the buffer C. After centrifugation at 16,000 × *g* for 30 min at 4°C, the pellet was dissolved in 500 μL distilled water. Final extracts were stored at -80°C prior to RNA extraction and analysis. To validate the NoV isolation process, another 10 g of digestive gland tissue spiked with murine norovirus was prepared. Briefly, the murine NoV culture supernatant of 1000 plaque-forming units was incubated for 30 min at room temperature with 10 g of digestive gland tissue in 50 mL of deionized water. After centrifugation at 4,500 × *g* for 30 min at 4°C, the pellet was collected and used for process control in the virus isolation.

### Viral RNA extraction

To extract the HNoV genome, up to 300 μL (≥ 140 μL) of each final concentrate was processed with a QIAamp viral RNA mini kit (Qiagen, Valencia, CA, USA) or with a Ribospin vRD (GeneAll, Seoul, South Korea). All extraction processes were performed according to the manufacturer’s instructions.

### Nucleic acid amplification

To minimize carryover contamination, all processes followed a previous description of quality assurance [24]. One-step reverse transcription polymerase chain reaction (RT-PCR) was performed using a Verso 1-Step RT-PCR ReddyMix kit (Thermo Fisher Scientific, Waltham, MA, USA) according to the manufacturer’s instructions with minor modifications. Briefly, the RNA extract (5 μL) from each concentrate was mixed with 2× 1-Step PCR ReddyMix, 2.5 μL RT enhancer, 40 μM forward/reverse primers (GIIFIM/GIIRIM for region C, GIIPF800M/GIICR1450 for the ORF1-ORF2 junction, or GII.FIM/GIICR1450 for the nearly complete VP1; Table 1) [24], and 1 μL of Verso Enzyme Mix. Deionized sterile water was added for a final reaction volume of 50 μL.

**Table 1:** Primers used in this study.

Genetic locus	Primer ID	Sequences^a^	Target region^b^
ORF2 (Region C, 0.3 kb)	GII-F1M	5′-GGGAGGGCGATCGCAATCT-3′	5049–5068
	GII-F3M	5′-TTGTGAATGAAGATGGCGTCGART-3′	5079–5102
	GII-R1M	5′-CCRCCNGCATRNCCRTTRTACAT-3′	5367–5389
ORF2 (1.6 kb)	GII-F1M	5′-GGGAGGGCGATCGCAATCT-3′	5049–5068
	GII-F3M	5′-TTGTGAATGAAGATGGCGTCGART-3′	5079–5102
	GIICR1450^c^	5′-ACCCARGMNTCAAAYCTRAART-3′	6622–6643
ORF1-ORF2 (1.0 kb)	GIIPF800M^c^	5′-GATGCWGAYTAYTCYMGNTGGGA-3′	4289–4311
	GIICR1450^c^	5′-ACCCARGMNTCAAAYCTRAART-3′	6622–6643
	GIIPF750M^c^	5′-CNGCHHTAGARRTNATGGT-3′	4341–4359
	GII-R1M	5′-CCRCCNGCATRNCCRTTRTACAT-3′	5367–5389

^a^ “H” = A or C or T “R” = A or G, “Y” = C or T, “V” = A or C or G, “W” = A or T, “N” = A or C or G or T

^b^ Corresponding nucleotide position of HNoV GII (GenBank ID: X86557)

^c^ Primers designed in this study

The RT-PCR conditions for region C were as follows: reverse transcription at 45°C for 30 min and 94°C for 5 min, followed by 35 cycles of amplification (94°C for 30 s, 55°C for 30 s, and 72°C for 90 s). A final extension was performed at 72°C for 7 min. The RT-PCR conditions for the ORF1-ORF2 junction and the nearly complete VP1 were as follows: reverse transcription at 45°C for 30 min and 94°C for 5 min, followed by 35 cycles of amplification (94°C for 1 min, 47°C for 1 min, and 72°C for 3 min). A final extension was performed at 72°C for 7 min. For virus isolation process control in clams (control murine NoV RNA from digestive gland tissue), primers and RT-PCR conditions followed a previous description [25].

Each of the first PCR products was used as a template for secondary nested PCR. The first PCR products (5 μL) were mixed with 10× buffer, 4 μL of 2.5 mM dNTPs, 50 μM forward/reverse primers (GIIF3M/GIIRIM for region C, GIIPF750M/GIIRIM for the ORF1-ORF2 junction, or GII.F3M/GIICR1450 for the nearly complete VP1; Table 1) [24], and 5 U of *Top* DNA polymerase (Bioneer, Daejeon, South Korea). Deionized sterile water was added for a final reaction volume of 50 μL. The secondary nested PCR conditions for region C were as follows: first denaturation at 94°C for 5 min, followed by 25 cycles of amplification (94°C for 30 s, 55°C for 30 s, and 72°C for 90 s). A final extension was performed at 72°C for 5 min. The secondary nested PCR conditions for the ORF1-ORF2 junction and the nearly complete VP1 were as follows: first denaturation at 94°C for 5 min, followed by 29 cycles of amplification (94°C for 1 min, 47°C for 1 min, and 72°C for 2.5 min). A final extension was performed at 72°C for 5 min. The secondary nested PCR conditions for control murine NoV (virus isolation process control of clams) followed the HNoV region C amplification described above (final amplicon size of 132 nt; forward primer MNV-2F: GTGGTTGTTGCCCTTGTA; reverse primer MNV-2R: CGACGCACGTCAAGAAGA).

### Cloning and sequence analysis

PCR amplicons having predicted size (310 nt for region C, 1050 nt for the ORF1-ORF2 junction, and 1560 nt for the VP1) were separated, purified, and cloned. Gene cloning was performed using a Mighty TA-cloning Kit (Takara, Kusatsu, Japan) and chemically competent DH5α (Enzynomics, Daejeon, South Korea). Based on the heterogeneity of NoV strains, six transformed colonies from each PCR amplicon were chosen for sequencing. Cloned genes were submitted for DNA sequencing (Macrogen, Seoul, South Korea) and analyzed using a 3730xl DNA analyzer (Thermo Fisher Scientific).

### NoV sequence preparation and phylogenetic analysis

The NoV sequences were first determined using the BLAST-n program (http://www.ncbi.nlm.nih.gov/) and confirmed using the Norovirus Genotyping Tool (http://www.rivm.nl/mpf/norovirus/typingtool) [26]. Using ClustalX (version 1.81), NoV sequences were aligned and subsequently processed using the Molecular Evolutionary Genetic Analysis version 6.0 (MEGA6) program.

The phylogenetic relationships of the NoV capsid region C or partial RNA dependent RNA polymerase region were determined using the neighbor-joining method with 1000 bootstrap replicates. The evolutionary distances were computed using the Kimura-2 parameter model (gamma shape distribution parameter of 4). In the maximum likelihood tree analysis for the NoV VP1 gene (1560 nt), GTR-gamma invariant model was selected as the evolutionary distance model (gamma shape distribution parameter of 4) after verification using MEGA6 and then analyzed with 1000 bootstrap replicates. The initial tree for the heuristic search was completed after applying the neighbor-joining method to a matrix of pairwise distances estimated by using the Maximum Composite Likelihood (MCL) method.

### Accession numbers of the isolated nucleotide sequences

The newly isolated original sequences in this study were deposited in the GenBank database with the following accession numbers: KT438785–KT438795, KT438799–KT438801, KT598020, KT598021, KT864684–KT864687, and KU687005–KU687039.

## Results

### Occurrence of HNoV in an estuarine environment in North Jeolla province, South Korea

**Fig 2:**
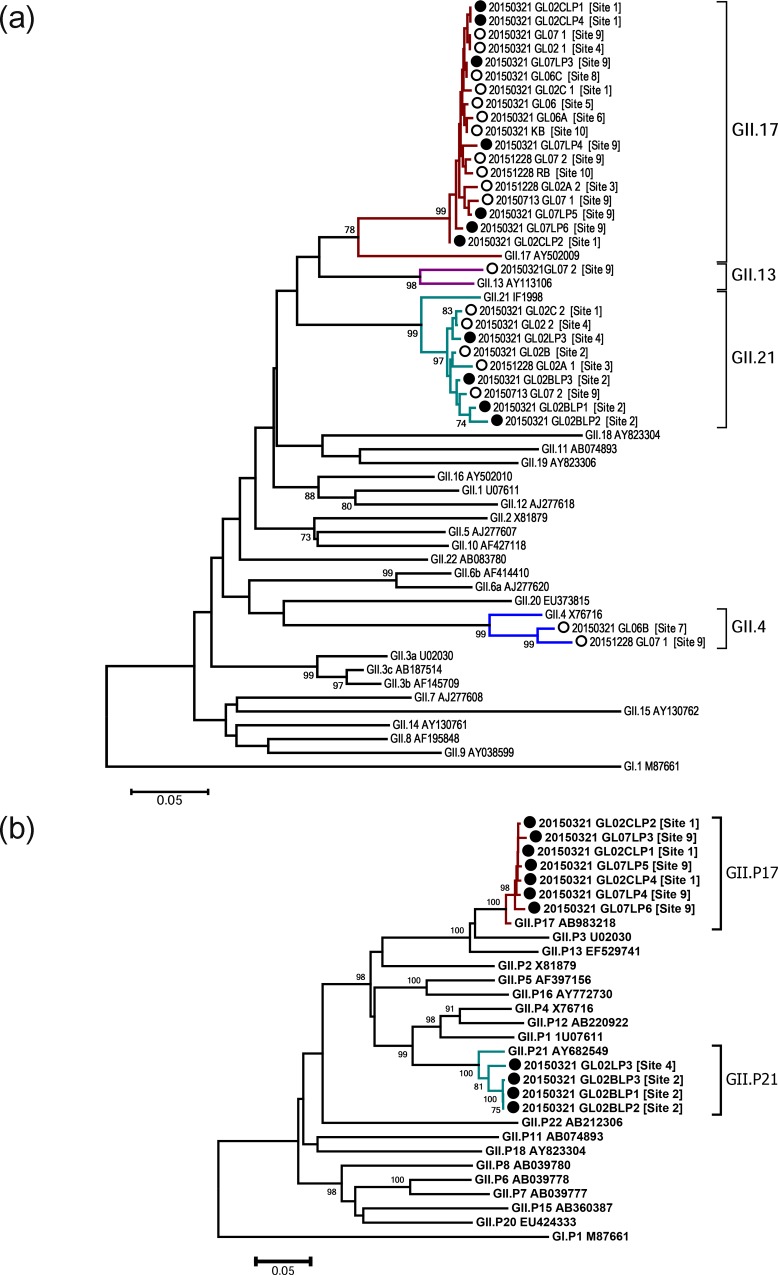
**Neighbor-joining tree based on the partial capsid gene (region C, 303 nt) (a) and partial polymerase gene (ORF1, 745 nt) (b) of norovirus nucleotide sequences from the study area.** The norovirus capsid region C nucleotide sequences (a), which were harvested from the study area, are indicated as open circles (○, sequences of the region C amplicons) and closed circles (●, region C sequences of the partial ORF1-ORF2 amplicons). The norovirus partial polymerase (ORF1) nucleotide sequences (b), which were harvested from the study area, are indicated as closed circles (●, ORF1 of the partial ORF1-ORF2 amplicons). The percentages of replicate trees in which the associated taxa clustered together in the bootstrap test (1000 replicates) are shown at the branches (bootstrap values of ≥70 are shown). The sampling site numbers are shown next to each sample label. Each sample label number on a tree indicates the sampling date (e.g., 20150321 = March 21, 2015).

Twenty-seven water sample concentrates obtained from two coastal streams and a neighboring waste water treatment plant (WWTP) at three distinct time points in 2015 were analyzed for NoV GII capsid region C (the 5′-end of major capsid protein 1, ORF2) by RT-PCR (Fig 1). The viral concentrate extracted from wild-growing short neck clams in a mud flat within the vicinity of the water sampling site 5 was also subjected to the same RT-PCR analysis. To determine genotypes of the NoV-related sequences based on the capsid region C sequences, phylogenetic analysis was carried out. Alignment of the 303-base nucleotide sequences enabled us to construct a neighbor-joining tree (Fig 2A). Including the clam samples, four different capsid region C genotypes of GII NoV, i.e., GII.4, GII.13, GII.17, and GII.21, were identified with different detection frequencies according to sampling site and season (Table 2). In March, nine of 10 sampling sites were GII-positive with a dominance of GII17: GII.17 (seven sites), GII.21 (three sites), GII.13 (one site), and GII.4 (one site). The positive cases of stream water samples and clam samples were clearly dependent on the season, whereas the treated sewage effluent (TSE) was the only type of sample that was positive regardless of the sampling time point.

**Table 2:** HNoV GII genotypes in water and clam samples in 2015.

Sample type	Sampling site	Sampling month				
		March^d^			July	December
		ORF2 Region C (0.3 kb)	ORF1-ORF2 (1.0 kb)	ORF2 (1.6 kb)	ORF2 Region C (0.3 kb)	ORF2 Region C (0.3 kb)
Stream water	1	GII.17, GII.21	GII.P17-GII.17	GII.21	-	-
	2	GII.21	GII.P21-GII21	GII.21	-	-
	3	-^e^	-	-	-	GII.17, GII.21
	4	GII.17, GII.21	GII.P21-GII.21	GII.17, GII.21	-	-
	5	GII.17	-	-	-	-
	6	GII.17	-	-	-	-
	7	GII.4	-	GII.4	-	-
	8	GII.17	-	-	-	-
TSE^a^	9 (WWTP^b^)	GII.13, GII.17	GII.P17-GII.17	GII.17	GII.17, GII.21	GII.4, GII.17
Clam	10 (Mud flat^c^)	GII.17	-	-	-	GII.17

^a^ Treated sewage effluent

^b^ Waste water treatment plant

^c^ In the vicinity of sampling site 5

^d^ Samples collected in March were used to amplify ORF1-ORF2 (1.0 kb) and ORF2 (0.3 kb or 1.6 kb).

^e^ Not detectable

### Phylogenetic analysis of ORF1 and ORF2

To identify the ORF1 (polymerase) genotype and reconfirm the ORF2 genotype, we next performed two additional long-RT-PCR analyses for the ORF1-ORF2 junction (1.0 kb) and the VP1 (1.6 kb, nearly full length of ORF2) region using the same samples collected in March. Of nine capsid region C-positive samples, we were able to amplify the ORF1-ORF2 junction sequences from four samples and the VP1 sequences from five samples (Table 2). Neighbor-joining phylogenetic analysis of the polymerase (3′-end of ORF1, 0.7 kb) and capsid region C (5′-end of ORF2, 0.3 kb) from the ORF1-ORF2 junction region (Fig 2A and 2B) revealed that all sequences from the study area were closely related to either one of two ORF1-ORF2 genotype categories: GII.P17-GII.17 and GII.P21-GII.21.

**Fig 3:**
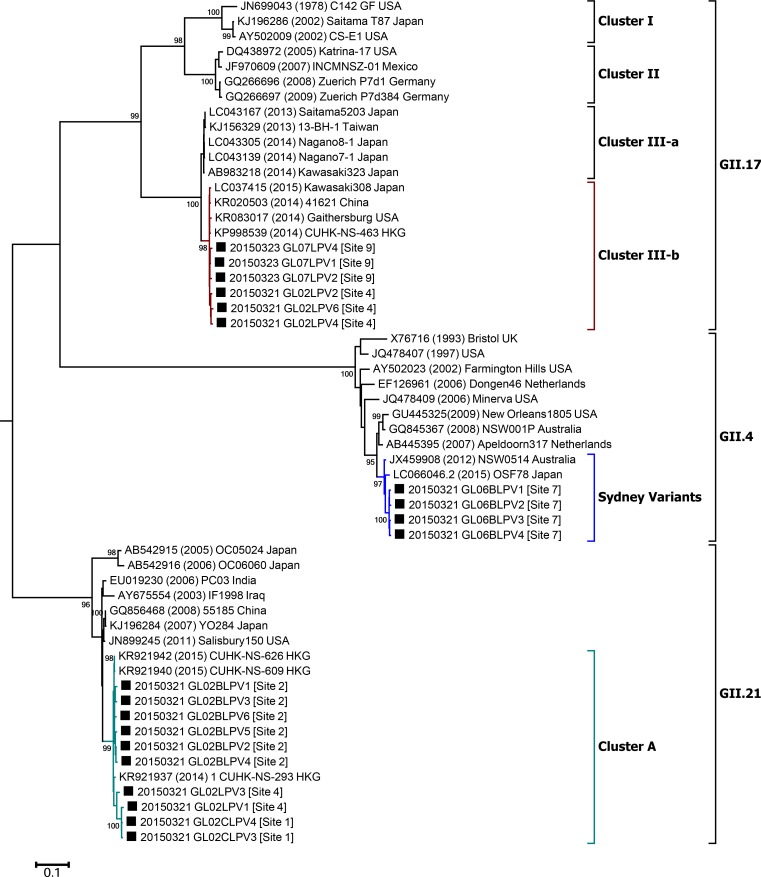
Phylogenetic tree of the partial VP1 (ORF2, 1,560 nt) in environmental samples determined by the maximum likelihood method. The VP1 nucleotide sequences of noroviruses harvested from the study area are indicated as closed squares (■). The sampling site numbers are shown next to each sample label. The percentages of trees in which the associated taxa clustered together are shown next to the branches (bootstrap values of ≥95 are shown). The tree is drawn to scale with branch lengths measured in the number of substitutions per site. Each sample label number on the tree indicates the sampling date (e.g., 20150321 = March 21, 2015). An outgroup reference strain (GI.1; GenBank ID: M87661) is not shown in this figure.

Maximum likelihood phylogenetic analysis based on the VP1 showed that sequences obtained from site 7 were closely related to the GII.4 Sydney 2012 pandemic variants. The non-GII.4 VP1 sequences in this study formed clusters with reference strains of GII.17 (cluster III-b) or with reference strains of GII.21 isolated recently (2014–2015; Fig 3).

Considering the phylogenetic trees for the polymerase (3′-end of ORF1, 0.7 kb), the capsid region C (5′-end of ORF2, 0.3 kb), and the VP1 region (1.6 kb), these results supported two conclusions: 1) the GII.17 sequences in the study area were closely related to the novel strains of GII.17, which were the most common strains isolated in recent years (2014–2015) in several Asian countries (e.g., China and Japan) [16–19]; 2) the GII.21 sequences of both North Jeolla province and Hong Kong isolated in 2014–2015 formed a phylogenetically independent sub-cluster from the former related strains of GII.21.

### Amino acid sequence variation in the major capsid protein 1 (VP1) of the GII.17 and GII.21 lineages

**Fig 4:**
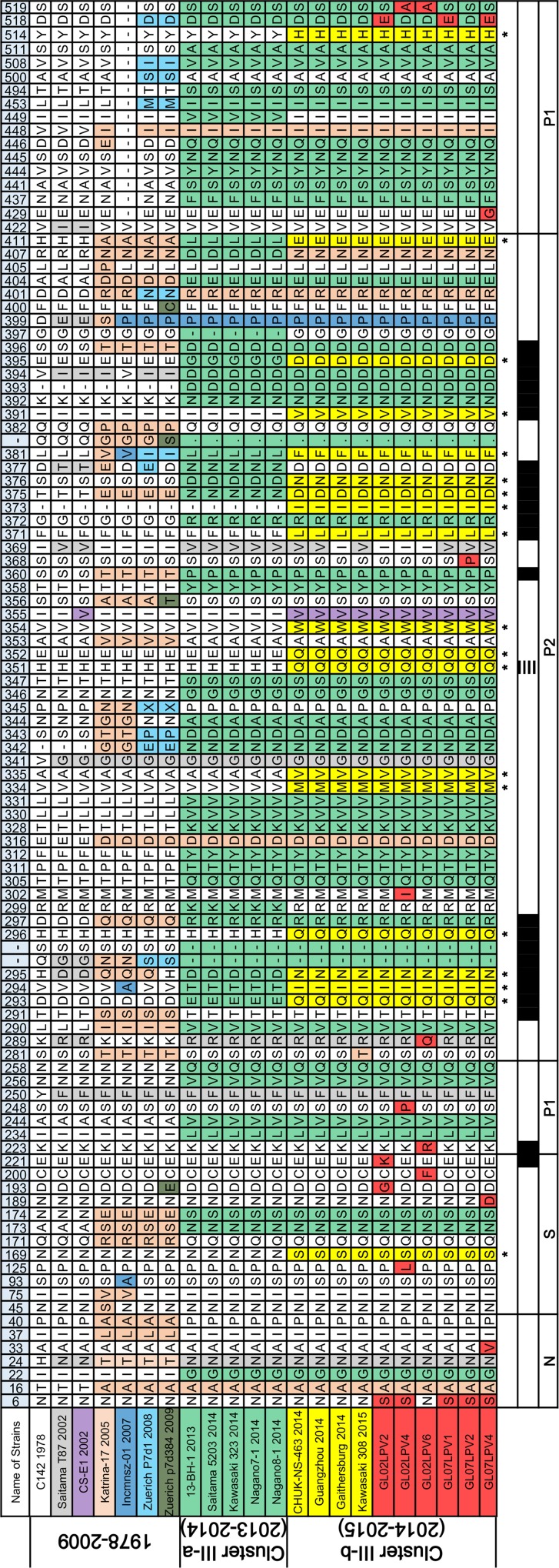
Amino acid sequence variation of VP1 among related strains of the GII.17 in South Korea. VP1 amino acid sequences of GII.17 from the study area were aligned with former related sequences. The VP1 amino acid sequences were aligned using DNAMAN, and heterogeneous sites were exported to a table, which were ordered according to time. Each color represents an amino acid change that occurred between strains or between sub-clusters. An amino acid position coinciding with the putative histo-blood group antigen (HBGA) binding sites of GII.4 pandemic variants (horizontal stripes) are indicated at the bottom of the alignment table. Black regions at bottom of tables indicate the putative B cell epitopes of GII.17. The asterisk (*) indicates commonly changed amino acid sites in the novel strains of GII.17 (cluster III-b) compared with former related strains.

**Fig 5:**
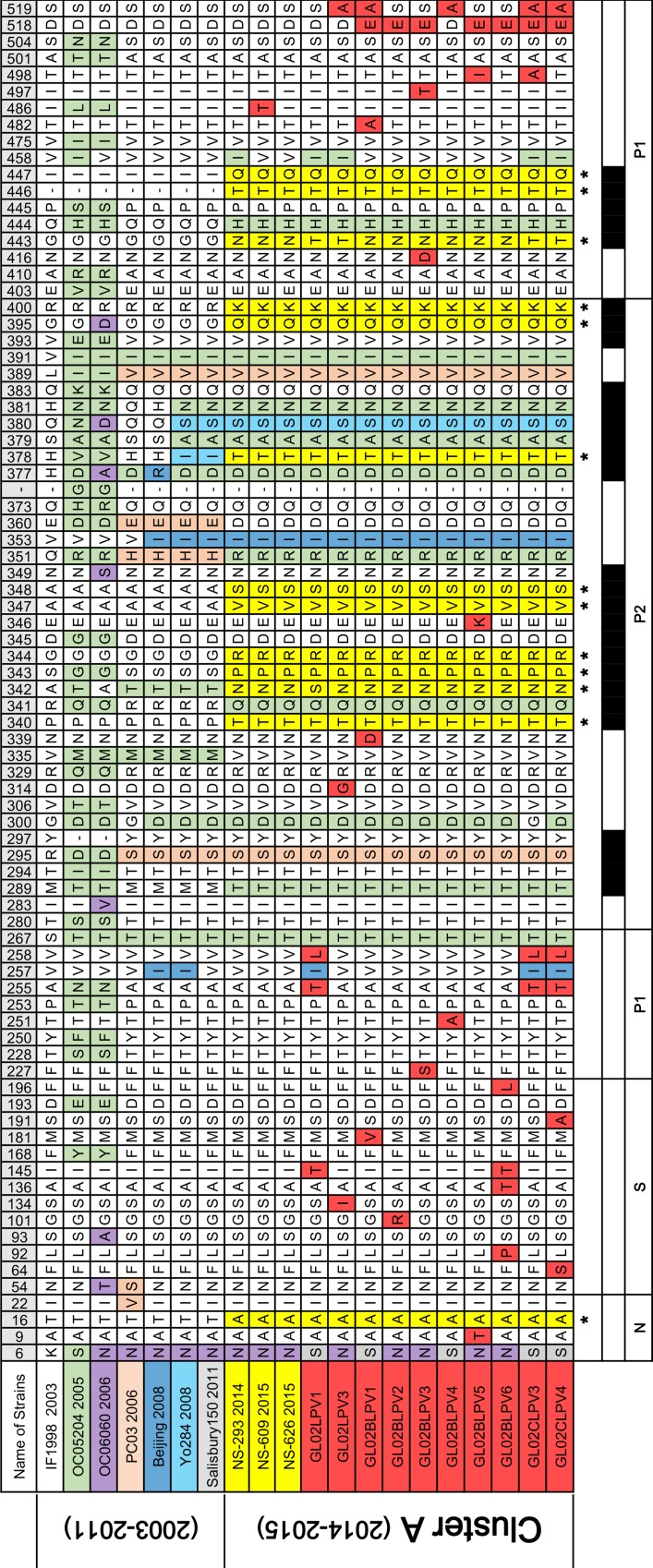
Amino acid sequence variation of VP1 among related strains of the GII.21 in South Korea. VP1 amino acid sequences of GII.21 from the study area were aligned with former related sequences. The VP1 amino acid sequences were aligned using DNAMAN, and heterogeneous sites were exported to a table, which were ordered according to time. Each color represents an amino acid change that occurred between strains or between sub-clusters. Black regions at bottom of table indicate the putative B cell epitopes of GII.21. The asterisk (*) indicates commonly changed amino acid sites in the novel strains of GII.21 (cluster A) compared with former related strains.

VP1 amino acid sequences of GII.17 and GII.21 identified in this study were compared with those of related lineages (Figs 4 and 5). Consistent with several recent reports describing the novel strains of GII.17 [17, 18], the amino acid sequence of the GII.17 VP1 region has changed recently, resulting in the designation of several sub-clusters (Fig 4). The GII.17 amino acid sequences from North Jeolla province were more similar to those of the novel VP1 sequences in China and Japan than to those of the other sub-clusters. Putative histo-blood group antigen (HBGA) binding sites [27, 28] and putative B cell epitopes [17] of VP1 corresponding to the changed amino acid sites in the GII.17 lineage are shown in Fig 4.

Interestingly, the VP1 alignment of the GII.21 lineage, including 10 sequences from North Jeolla province and three sequences from Hong Kong, showed that 13 commonly changed sites were present, as compared with formerly related strains (Fig 5). Of the 13 commonly changed sites, most of them (9/13) were located within the protruding P2 domain. Web-based B-cell epitope prediction programs (BepiPred 1.0 and DiscoTope 2.0) for GII.21 showed that the 12 of 13 commonly changed sites corresponded to four of the six putative B cell epitope regions. To present changed amino acid regions only, one conserved epitope region (P217 to P226) within sub-clusters was excluded from Fig 5.

## Discussion

In this study, environmental samples were collected from eight water sampling sites in two streams and one site in a WWTP as well as one site on a mud flat to be representative of the coastal environment of North Jeolla province, South Korea. To prevent over-generalization of HNoV GII genotype prevalence in estuarial samples collected in only one season, we investigated water and clam samples in March, July, and December of 2015. Because norovirus genotypes detected in streams are often representative of endemic GII cases of neighboring human dwellings [21], we specified multiple sampling sites along with the study streams. Raw sewage flowing to the WWTP (site 9) originated from a human community that was partially outside of the study area. Accordingly, a dominant norovirus genotype in TSE could complement generalization of endemic norovirus genotypes in the study area. TSE and stream water containing HNoV flow into the marine environment, which causes accumulation of norovirus in estuarine bivalves [22]; thus, we collected wild-growing short neck clams to confirm dominant norovirus genotypes in the study area. In total, 27 water samples from two coastal streams and a WWTP plus three clam samples from a neighboring mud flat were analyzed for HNoV GII contamination in this study. The identified NoV sequences in those samples were genotyped as GII.4, GII.13, GII.17, and GII.21.

Of the total 30 environmental samples, GII.4 was detected in only two water samples (Table 2). The GII.4 VP1 sequences (1.6 kb) from sampling site 7 were phylogenetically related with pandemic GII.4 variants. Owing to the rapid gene evolution rate and ORF1-ORF2 recombination, NoV GII.4 has effectively escaped herd immunity [29–31]. The GII.4 capsid has high mutation rates, particularly throughout the P2 sub-domain of the major capsid protein [32]. Changing of amino acid sequences in the major antigenic determinants known as blockade epitopes and of binding profiles to the HBGA in the P2 domain have been effective mechanisms for escaping herd immunity [29, 33–38]. The resulting GII.4 pandemic variants, such as US96 1990, Farmington Hills 2002, Hunter 2004, Den Haag 2006b, New Orleans 2009, and Sydney 2012, have rapidly replaced the former dominant strain [14]. As observed worldwide, GII.4 in South Korea was the most common causative genotype for acute gastroenteritis until 2013 [39].

In this study, however, dominance of GII.17 in water and clam samples over GII.4 was observed, and this may reflect the prevalence of GII.17 throughout the study period. Moreover, a recent report regarding NoV monitoring in nationwide coastal waters in South Korea partly supports dominance of GII.17 in 2015 [40]. Because NoV in clinical samples frequently reflects the genotype occurrence in the adjacent environment [21, 22], we can assume that GII.17 NoV is the most common genotype in patients in the study area. However, the lack of clinical data in North Jeolla province in 2015 prevents an actual determination that GII.17 is the main causative genotype for acute gastroenteritis in the study area. Frequent detection of GII.17 in environmental samples of this study area immediately reminded us of recent reports from China and Japan. In 2014–2015, novel GII.17 variants replaced the prevalent GII.4 sequences in Kawasaki prefecture in Japan and in Guangdong, Huzhou, and Jiangsu provinces in China [16–19]. Analysis based on amino acid sequences revealed that the new strains of GII.17 in China and Japan exhibit alterations in amino acid sequences in putative HBGA binding sites and in predicted antibody binding regions compared with former GII.17 strains [17, 18].

Furthermore, sequence analysis of the ORF1-ORF2 junction region and the VP1 gene obtained from the GII.17 NoVs in North Jeolla province enabled us to demonstrate that both sequences were closely related to the GII.P17-GII.17 strains of recent China/Japan. These observations therefore suggest two possibilities: first, GII.17 NoVs overwhelmingly prevalent in South Korea are the novel GII.17-related members that were identified in China and Japan very recently. Second, the antigenic characteristics of the novel GII.17 variants may have acquired the highest fitness in East Asia.

The GII.21 sequences (region C) in this study area showed the second highest prevalence (Table 2). Interestingly, the VP1 (1.6 kb) sequences of the GII.21 identified in this study and those of the GII.21 Hong Kong strains (GenBank registered in 2014–2015) formed a sub-cluster distinct from that of former GII.21 strains. The putative B-cell epitopes on VP1 of the new sub-cluster of GII.21 exhibited different amino acid sequence characteristics compared with those of the former strains. Until recently, GII.21 had not been observed frequently in either environmental samples or clinical cases; thus, this strain was not considered a major public health issue worldwide. Although we could only use a limited amount of amino acid sequence data, it should be noted that the P2 region of VP1 sequence in the GII.17 and GII.21 lineage (Figs 4 and 5) appears to have been altered similarly to that in the GII.4 lineage [14]; thus, these novel non-GII.4 variants may constitute a future threat to public health along with GII.4.

Inter- or intra-genogroup recombination between ORF1 and ORF2 has accelerated the evolution of NoV, allowing the capsid (particularly in GII.4) to replicate fast and to have a high mutation rate owing to the presence of the ORF1 genotype [29]. The GII.17 capsid from recent outbreaks was associated with an ORF1 genotype, GII.P17 [17–19, 41]. This GII.P17 genotype may support the GII.17 lineage and allow it to occupy the niche of other genotypes. In general, the capsid sequences of GII.13 and GII.21 NoVs occurred in combination with various ORF1 genotypes: i.e., GII.P12, GII.P13, GII.P16, GII.P21, GII.Pg, and GII.Pm, according to the Norovirus Genotyping Tool analysis (http://www.rivm.nl/mpf/norovirus/typingtool; data not shown) [42–47]. However, we could not detect the ORF1 gene associated with the GII.13 capsid gene; therefore, whether the GII.13 identified in this study is a new ORF1-ORF2 genotype recombinant was not determined (Table 2). Because ORF1-ORF2 recombination often arises after outbreaks caused by multiple ORF1-ORF2 genotypes, emergence of the most contagious ORF1-ORF2 recombinant among GII.Pe-GII.4, GII.P17-GII.17, and GII.P21-GII.21 may be possible in the future.

It is noteworthy that clams in the bay were affected by the same HNoV genotype detected within neighboring coastal streams and the WWTP. Indeed, GII.17 was dominant in both the clams and water samples. Although we did not describe these findings in this report, we detected the GII.P17-GII.17 sequences (GenBank ID: KT598020 and KT598021) from wild-growing clams that had been collected in the study area in February 2015. Therefore, it is possible that the novel GII.17 variants had already been circulating as an endemic strain in South Korea before March 2015. This possibility is strongly supported by a recent report of a noticeable increase in GII.17 in stool samples of hospitalized patients during first half of 2015 in Seoul, South Korea [48]. Moreover, increased detection of GII.17 NoV in acute gastroenteritis in children after November 2014 partly supports this possibility [49]. In addition to the two streams, the study area contained several other coastal streams that were not associated with any sanitation facilities, which suggests that HNoV contamination in the bay may have occurred on a large scale.

In summary, our findings demonstrate that non-GII.4 lineages could also become major genotypes, although the duration of predominance is unpredictable. Thus, to prepare for the possibility that non-GII.4 capsid lineages may trigger a new pandemic, future public health strategy must include vaccine development also targeting non-GII.4. Our future studies of the novel variants of GII.17 and GII.21 will focus on whether the predominance of these viruses is sustained for an extended period in broader environmental regions of South Korea.
